# Random Sampling of Squamate Reptiles in Spanish Natural Reserves Reveals the Presence of Novel Adenoviruses in Lacertids (Family Lacertidae) and Worm Lizards (Amphisbaenia)

**DOI:** 10.1371/journal.pone.0159016

**Published:** 2016-07-11

**Authors:** Leonóra Szirovicza, Pilar López, Renáta Kopena, Mária Benkő, José Martín, Judit J. Pénzes

**Affiliations:** 1 Institute for Veterinary Medical Research, Centre for Agricultural Research, Hungarian Academy of Sciences, 21 Hungária krt., Budapest, H-1143, Hungary; 2 Departamento de Ecología Evolutiva, Museo Nacional de Ciencias Naturales, C.S.I.C, José Gutiérrez Abascal 2, E-28006, Madrid, Spain; University Claude Bernard Lyon 1, FRANCE

## Abstract

Here, we report the results of a large-scale PCR survey on the prevalence and diversity of adenoviruses (AdVs) in samples collected randomly from free-living reptiles. On the territories of the Guadarrama Mountains National Park in Central Spain and of the Chafarinas Islands in North Africa, cloacal swabs were taken from 318 specimens of eight native species representing five squamate reptilian families. The healthy-looking animals had been captured temporarily for physiological and ethological examinations, after which they were released. We found 22 AdV-positive samples in representatives of three species, all from Central Spain. Sequence analysis of the PCR products revealed the existence of three hitherto unknown AdVs in 11 Carpetane rock lizards (*Iberolacerta cyreni*), nine Iberian worm lizards (*Blanus cinereus*), and two Iberian green lizards (*Lacerta schreiberi*), respectively. Phylogeny inference showed every novel putative virus to be a member of the genus *Atadenovirus*. This is the very first description of the occurrence of AdVs in amphisbaenian and lacertid hosts. Unlike all squamate atadenoviruses examined previously, two of the novel putative AdVs had A+T rich DNA, a feature generally deemed to mirror previous host switch events. Our results shed new light on the diversity and evolution of atadenoviruses.

## 1. Introduction

Adenoviruses (AdVs) are non-enveloped, double-stranded DNA viruses with icosahedral virions of 70−100 nm in diameter. AdVs commonly occur in humans and mammalian animals as well as in other representatives of vertebrates worldwide. Each host may harbor various number of AdV types classified by serology. The family *Adenoviridae* is divided into five genera presently [1]. The basis for the genus classification was originally the host origin, especially for the two conventional genera, *Mastadenovirus* and *Aviadenovirus*. The genus *Ichtadenovirus* was established to include the single fish AdV isolate known to date [2]. Two additional novel genera contain AdVs originating from distantly related hosts. *Siadenovirus* includes a frog isolate [3] along with an increasing number of AdV types isolated from, or described in, birds [4]. Fatal siadenovirus infection has been described among captive tortoises as well [5]. First members of the genus *Atadenovirus* have been isolated from cattle and other ruminants as well as birds, however, targeted studies aiming at the clarification of the host origin of this virus lineage led to the recognition of squamate reptiles as the vertebrate group, with which atadenoviruses most probably co-speciated [6–8].

The presence of AdVs in squamate hosts has been observed by serology, light and electron microscopy, and by PCRs. A consensus, nested PCR system, targeting a conservative short (approx. 300 bp) fragment of the DNA dependent DNA polymerase gene (*pol*) proved to be very sensitive and efficient in detection of AdVs in reptiles [8]. Various squamate groups turned out to harbor different AdVs. These include members of the Agamidae, Chamaeleonidae, Helodermatidae, Iguanidae, Scincidae, and Varanidae families, as well as those of the infraorder Gekkota and several snake groups, such as boids [9–34].

Clinical signs ascribed to AdV infection in squamate reptiles may vary and most commonly include lethargy, anorexia, enteritis, pneumonia, as well as neurological signs such as opisthotonus [33,35,36]. As infection without any clinical signs has also been observed, the pathological role of AdVs in lizards and snakes still remains unclear [14,28,37].

The majority of our knowledge on AdVs of reptiles comes from captive pet animals, most often produced by licensed breeders in North America and Europe. Data concerning the prevalence of AdVs in free-living reptiles are still rather limited [38,39].

In the present study, our aim was to estimate the prevalence and diversity of AdVs in specimens of free-living native reptiles, captured alive at the Spanish national park Guadarrama Mountains and in Northern Africa for the purpose of ecological or behavioral studies. We used PCR with consensus nested primers, DNA sequencing and phylogeny inference for the preliminary characterization of the newly-detected AdVs.

## 2. Materials and Methods

### 2.1. Samples

During April-May 2013, we made field work at several sampling sites at different elevations in the Guadarrama Mountains National Park (Central Spain) and the surroundings. We captured by noosing Iberian green lizards (*Lacerta schreiberi*) at ‘Valle de La Fuenfría’ (40°44′ N, 4°02′W) and Carpetane rock lizards (*Iberolacerta cyreni*) at ‘Puerto de Navacerrada’ (40°47′ N, 4°00′W). We also lifted stones and captured Iberian worm lizards (*Blanus cinereus*) by hand at `La Dehesa de la Golondrina´, near Navacerrada village (40°43´N, 04°01´W).

During two weeks in March 2013, we conducted field work also at the Chafarinas Islands Nature Reserve, a small archipelago located in the south western area of the Mediterranean Sea (35°11’N, 2°25’W). These islands are located 4.6 km off the northern Moroccan coast (Ras el Ma, Morocco) and 50 km to the east of the Spanish city of Melilla. Here, we lifted stones and captured by hand live amphisbaenians, namely checkerboard worm lizards (*Trogonophis wiegmanni*), Chafarinas’ skinks (*Chalcides parallelus*), ocellated skinks (*Chalcides ocellatus*), Moorish geckos (*Tarentola mauritanica*) and Vaucher’s wall lizards (*Podarcis vaucheri*) (Table 1).

**Table 1:** Results of PCR screening for the presence of adenoviruses by amplifying an approx. 300-bp-long fragment of the DNA-dependent DNA-polymerase gene.

Species (family)	Collection site	Number of samples screened	Number of positive samples	Percentage
**Iberian worm lizard *(Blanus cinereus*)(Amphisbaenidae)**	**GM**	**18**	**9**	**50%**
Checkerboard worm lizard *(Trogonophis wiegmanni*)(Trogonophidae)	CI	102	0	0%
Ocellated skink *(Chalcides ocellatus)*(Scincidae)	CI	10	0	0%
Doumergue’s skink *(Chalcides parallelus)* (Scincidae)	CI	19	0	0%
**Carpetane rock lizard *(Iberolacerta cyreni)* (Lacertidae)**	**GM**	**36**	**11**	**30.6%**
**Iberian green lizard *(Lacerta schreiberi)* (Lacertidae)**	**GM**	**59**	**2**	**3.4%**
Vaucher’s lizard (*Podarcis vaucheri*)(Lacertidae)	CI	17	0	0%
Common wall gecko (*Tarentola mauritanica)* (Phyllodactylidae)	CI	57	0	0%
**Total**		**318**	**22**	**6.9%**

CI: Chafarinas Islands Nature Reserve (North Africa); GM:–Guadarrama Mountains National Park (Central Spain). The AdV-positive species are highlighted in bold.

Immediately after capture, animals were taken to the laboratory and cloacal swabs were collected within sterile circumstances the same day. A total of 318 cloacal swabs were stored frozen (at -20°C) until DNA extraction. All animals were released in good conditions at the capture sites at the end of the study, approximately two weeks after capture (exact release dates varied between species and individuals). The captures and sampling procedures enforced all the current European laws and ethical principles and were performed under license (permit number: 10/024398.9/13) from the Environmental Organism of Madrid Community (“Area de Conservación de Flora y Fauna. Dirección General del Medio Ambiente. Consejería de Medio Ambiente y Ordenación del Territorio”) and by the Organismo Autónomo de Parques Nacionales.

### 2.2. DNA extraction

For nucleic acid extraction, 1 ml of 1X TE buffer (10 mM Tris-HCl, 1 mM EDTA, pH 8.0) was added to the vials and the swabs were soaked overnight. During the next day, microcentrifuge tubes, containing the swabs in TE buffer, were continuously vortexed at 40 Hz. DNA was purified from 100 μl suspension, using the method described by Dán et al. [40] with slight modifications. We added 4 μl proteinase-K (20 mg/ml) and 10 μl sarcosyl (10%) solution to the mixture and incubated them at 55°C for overnight digestion in a thermomixer. This was followed by the addition of 300 μl guanidine-hydrochloride (8 M) and 20 μl ammonium-acetate (7.5 M) solution. The mixture was incubated for one hour with gentle mixing in every 15 min. The nucleic acids were precipitated by the addition of absolute ethanol (-20°C). After centrifugation, the pellet was washed with 70% ice-cold ethanol, and spun again. After a short drying, the DNA was dissolved in 50 μl of nuclease-free water.

### 2.3. Sample screening

To check the presence of adenoviral DNA, a very sensitive consensus nested PCR, targeting a highly conserved region of the adenoviral DNA-dependent DNA polymerase gene, was applied [8]. The amplification product was an approximately 320-bp-long fragment. After primer removal, a 269−278-nucleotide-long sequence was to be obtained supposedly from every member of the family *Adenoviridae*. The PCRs were performed in 50 μl final volume consisting of the REDTaq^®^ ReadyMix™ (Sigma-Aldrich^®^, Saint Louis, MO, USA) polymerase enzyme according to the manufacturers’ recommendation. The thermal profile of the PCR was identical to that described originally [8] except the denaturation steps that were set at 95°C.

We used another set of consensus primers, designed at our laboratory, to amplify an approx. 900-bp-long fragment from the conserved central part of the adenoviral genome [41]. The forward primer (5’-AATRTNCCYTHTGTTGCAGATCACG-3’) was designed to anneal close to the stop codon of the penton base gene. The sequence of the reverse primer (5’-CCRCARTGSGGNGCTARKC-3’) was taken from the beginning of the pVI gene of atadenoviruses. The PCR program consisted of an initial denaturation step at 95°C for 5 min, followed by 45 cycles of denaturation at 95°C for 30 sec, annealing at 46°C for 1 min, and elongation at 72°C for 1 min. The final elongation was at 72°C for 5 min.

The prevalence of AdVs was estimated as the percentage of positive samples out of the total number of individuals of a given species collected and screened from a given collection site.

The PCR products were purified and sequenced directly on both strands. We performed the sequencing reactions using the BigDye^®^ Terminator v3.1 Cycle Sequencing Kit (Life Technologies Corporation^®^, Carlsbad, CA, USA), and sent them for electrophoresis by a commercial service at Baygen Institute (Szeged, Hungary) on an ABI PRISM 3100 Genetic Analyzer (Life Technologies Corporation^®^, Carlsbad, CA, USA).

### 2.4. Sequence analysis and phylogenetic inference

For identification and comparison of the nucleotide (nt) sequences, the BLAST algorithms at the NCBI website were used. Sequence editing and assembly was performed by applying the Staden Sequence Analysis Package [42] with occasional manual corrections.

We performed phylogenetic calculations with amino acid (aa) sequences derived from the nucleotide (nt) sequence of the PCR products. The homologous protein fragments were collected from the GenBank with the use of the BLASTp application. Several representatives from each AdV genus were included. Multiple aa alignments were prepared using the Clustal X version 2 program [43].

Model selection was performed by the ProtTest version 2.3 [44]. Best model was applied based on the Akaike (AIC) and Bayes (BIC) information criterion. Guide tree was constructed using PHYLIP 3.69 (Protdist with JTT then Fitch with global rearrangements). We performed the maximum likelihood analysis using the PhyML 3.0 online platform, with the results of the model selection included (http://www.atgc-montpellier.fr/phyml/). The reliability of tree topology was tested by performing bootstrap analysis as well as by aLRT-Shimodaira-Hasegawa-like test. Phylogenetic trees were visualized using the FigTree v1.3.1. software (http://tree.bio.ed.ac.uk/software/figtree/).

## 3. Results

Out of the 318 samples, 22 proved to be positive, which corresponded to an overall positivity rate of 6.9% however, the AdV prevalence values for the individual populations showed great differences, inasmuch as among the Iberian green lizards it was just 3.4%, whereas among the Carpetane rock lizards and Iberian worm lizards was 30.5% and 50%, respectively (Table 1). All positive samples were from animals captured in Central Spain, whereas every sample collected on the Chafarinas Islands was found negative. Out of the five squamate families, involved in the survey, merely two, the Amphisbaenidae and the Lacertidae were represented among the positive samples.

After removal of the primer sequences, the *pol* fragments had a total length of 272 bp coding for 90 aa. Analysis of the sequences revealed the 22 positive samples to contain three hitherto undetected putative AdVs. The sequence data obtained from these viruses was deposited to GenBank and assigned to accession numbers (KT950885-KT950888). None of the novel adenoviral sequences demonstrated higher aa identity percentage than 74% throughout the BLAST homology searches.

**Fig 1:**
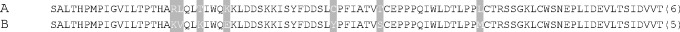
**Two (A and B) sequence variants of the putative lacertid adenovirus 2 found in 6 and 5 Carpetane rock lizards, respectively.** The divergent amino acids are highlighted with gray background.

One of the newly-detected AdVs originated from the swabs of Iberian worm lizards (*Blanus cinereus*), a native amphisbaenian of the Iberian Peninsula. It is noteworthy that 50% of the samples of this animal species were positive, and the sequences in all the 9 samples proved to be identical, even at nt level. We named this putative virus amphisbaenian AdV-1 (AmAdV-1). A second novel AdV was detected from the swabs of two Iberian green lizards (*Lacerta schreiberi*), also resulting in sequences completely identical even at nt level. This putative virus was designated lacertid AdV-1 (LaAdV-1). Both individuals were males with nuptial coloration and displayed mating behavior. A third, hitherto unknown *pol* sequence was obtained from 11 animals belonging to another lacertid species, the Carpetane rock lizard (*Iberolacerta cyreni*) with a positivity rate exceeding 30%. Interestingly, the AdV sequences obtained from these lizards showed variations so that two hypothetic genotypes exhibiting almost 8% aa sequence divergence were recognized (Fig 1). The virus was named lacertid AdV-2 (LaAdV-2).

We attempted to amplify further sequences of the genomes of the three novel reptilian AdVs. This was, however, successful only in case of two samples positive for the AmAdV-1. The resulting 801-bp-long product encompassed the 3’ part of the penton base gene, and the complete pVII and pX genes. The sequence was submitted to GenBank under the accession number of KT932964. The complete pVII gene of AmAdV-1 is predicted to encode a protein of 139 aa. The core protein pX, being the smallest adenoviral protein, consisted only of 61 aa, being the shortest such protein of atadenoviruses thus far.

**Fig 2:**
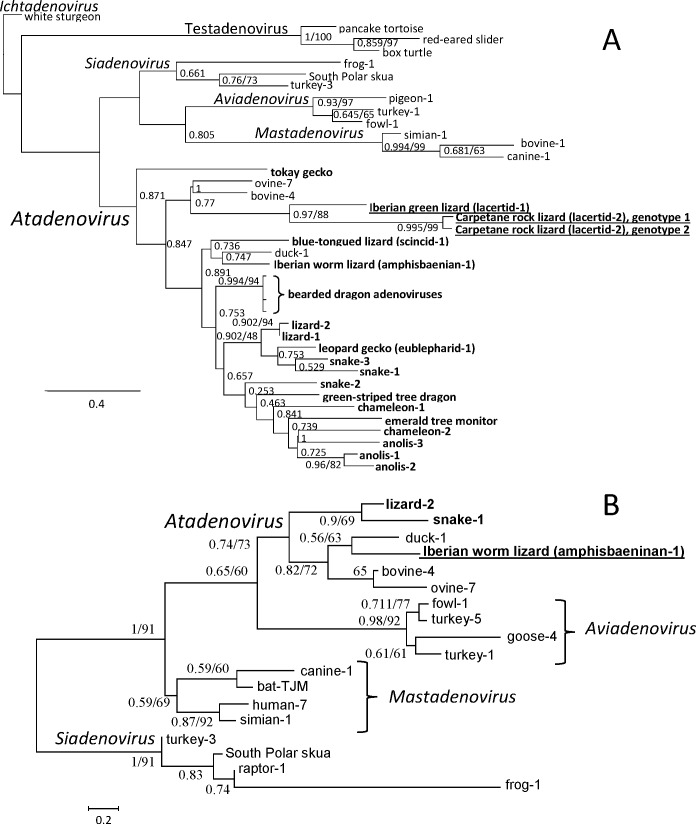
**Phylogeny reconstructions based on (A) the partial amino acid sequence of the DNA-polymerase (92 amino acids, maximum likelihood, LG+I+G, α = 0.63, ρinv = 0.17) and (B) the complete pVII ORF amino acid sequence (92 amino acids, maximum likelihood, RtREV+I+G+F, α = 1.095 ρinv = 0.039).** Shimodaira-Hasegawa-like support values (SH) and bootstrap values are shown as node labels. SH values are shown if higher than 0.5, whereas bootstrap values only over the value of 50. Squamate adenoviruses are highlighted in bold, novel adenoviruses detected in this study are underlined.

Phylogeny inference, based on the aa sequences derived from the PCR-amplified *pol* fragments was suitable for the presentation of the clear monophyletic separation of every proposed and accepted adenoviral genus [8,25,33]. The tree shown in Fig 2A was prepared using the LG substitution model as the most appropriate based on both the AIC and BIC values. Gamma distribution and proportion of invariable sites were also included in the applied model. All three novel reptilian AdVs clustered with members of the genus *Atadenovirus*. Within the genus, lacertid AdVs displayed a rather well-supported monophyly. Surprisingly, the amphisbaenian AdV clustered with duck AdV-1 (DAdV-1). However, supporting values, regarding this and some other nodes, cannot be considered significant. A second phylogenetic tree, shown in Fig 2B, was constructed based on the derived aa sequences of the complete pVII ORF, including the pVII aa sequence of the AmAdV-1. Both the AIC and BIC values suggested RtREV, a substitution model developed to describe the rapid evolution of retroviruses. Gamma distribution as well as proportion of invariable sites and aa frequencies were included. By this tree, monophyly of each genus was further supported as well as the position of AmAdV-1 clustering with DAdV-1, however, support values still remained relatively low.

The G+C content of the short *pol* sequences determined in this study showed remarkable variations. The individual values are presented in Table 2. While the nt composition of the LaAdV-2 fragment was within the range of equilibrium, that of LaAdV-1 and AmAdV-1 sequences seemed to be biased towards A+T, similarly to atadenoviruses of non-reptilian hosts [1].

**Table 2:** G+C content of the short (PCR-amplified) fragments of the DNA polymerase gene and the complete genome (if available) of selected atadenoviruses.

Name of the virus	G+C content of the pol fragment (~300 bp)	G+C content of the complete genome
Scincid-1	52.9%	N/A
Snake-1	51.1%	55.3%
Eublepharid-1	50%	N/A
Lizard-2	45.8%	**44.2%**
Lacertid-2	45%	N/A
Amphisbaenian-1	**41.2%**	43.13*
Lacertid-1	**37.6%**	N/A
Duck-1	47.4%	**43%**
Bovine-4	**37.1%**	**38.5%**
Bovine-6	**36%**	**35.1%**
Ovine-7	**32.4%**	**37.5%**
Possum-1	**38.2%**	40.4%*

Non-squamate atadenoviruses, supposed to have switched hosts, are separated from the squamate atadenoviruses by a thick line. Values lower than 45% are highlighted in bold. The names of the newly-detected putative adenoviruses are underlined. Values, marked with an asterisk (*) are deduced from incomplete genome sequences.

## 4. Discussion

Prior to this study, PCR-detection and sequencing of AdVs in samples of deceased or live captive reptiles have been reported several times [8,25,29,33]. However, our study is the first survey aiming at the assessment of the prevalence and diversity of AdVs in free-living wild reptiles in Europe and North Africa. The overall positivity rate of 6.9% can be considered rather low especially in comparison with previous findings. For example, in a survey performed in Germany, an infection rate of 36.1% has been reported [25]. However, their sample collection, from about 70 dead or live captive snakes and lizards, predominantly consisted of animals that had been previously suspected to have adenovirus infections. Also in Germany, the prevalence of neutralizing antibodies against different reptilian AdVs was found to be 33.8% and 44.9%, among lizards and snakes, respectively, belonging to various species [39]. However, because of the different target and sensitivity, the discrepancy between the results of serological tests or PCR detection is not unprecedented. Moreover, the role of innate antibodies in false positive serology results should also be considered [45,46].

The interpretation of the low overall positivity rate, observed in our study, is challenging especially because of the uneven distribution. From animals, sampled at the Chafarinas Islands, we could not derive any AdV sequences at all, although the majority (two thirds) of the samples were collected here. Nevertheless, even if our data are preliminary, the failure to detect adenoviral DNA in the significant number (205) of samples examined may suggest a significantly lower prevalence if not complete absence of AdVs in reptiles of the Chafarinas Islands natural reserve, which is a considerably good news from a conservationist point of view. The combined number of positive samples of animals examined at the Guadarrama Mountains, was 22 representing an almost 20% of positivity. A strikingly high infection rate (50%) was demonstrated among the Iberian worm lizards. Controversially, all of these individuals appeared to be healthy, showing no clinical signs or behavioral anomalies. The same applies for the positive Carpetane rock lizards as well. These findings corroborate that AdV infection without notable disease or clinical signs may be present in different vertebrate hosts including adult squamate reptiles [36,37,47].

As a result of this study, we discovered the presence of three, hitherto unknown AdVs. However, for their ultimate confirmation, acquisition of the sequences of additional genes or, preferably, of the entire genome would be indispensable. Our first attempts were fruitful with the AmAdV-1, which is not only the very first AdV ever detected in an amphisbaenian host, but only the second virus, after a parvovirus, found in any member of this cryptic, yet ecologically and evolutionally important group of squamates [48].

The first members of the genus *Atadenovirus* have been recognized in ruminant mammals and birds. These viruses have shown irregular properties, and have later been found to possess genomic DNA with very low G+C content, hence the name of the genus [49]. During the first adenovirus phylogeny reconstructions, their large distance from members of the other two genera (*Mastadenovirus* and *Aviadenovirus*) has been discovered. The hypothesis on the possible co-speciation and co-evolution of AdVs and their respective vertebrate hosts was based on the similarity between the topology of phylogenetic trees of AdVs and their hosts. Initially, the lineage of atadenoviruses had been thought to belong to reptiles in general [6]. However, targeted examinations revealed that atadenoviruses are prevalent among squamate reptiles only, whereas non-squamate reptiles seem likely to have their separate AdV lineages [50]. Interestingly however, the G+C content of the partial or full genomic DNA sequences obtained from snake and lizard AdVs, has been found usually in a non-biased range of 45−55% [8,25,51].

The atadenoviruses found in some mammals and ruminants are now hypothesized to have switched hosts and the higher A+T content in their DNA might result from the adaptation process to the new host [52]. Indeed, such phenomenon has been reported with other viruses recently [53]. The overall genomic base-composition bias is mirrored even in the short *pol* gene fragments [8,25]. Interestingly, the phylogeny analysis clearly assigned our three newly-detected putative AdVs to the genus *Atadenovirus*, the G+C content can be considered balanced in case of LaAdV-2 exclusively (45% as shown in Table 2). Although LaAdV-1 clustered with LaAdV-2 as a monophyletic branch, the G+C content in its *pol* sequence is only 37.6%. This contradiction is difficult to resolve, as the mechanisms, leading to this bias are poorly understood. The selection pressure, due to the ability of the innate immune system to recognize non-methylated CG dinucleotides, could provide a plausible explanation [54,55]. From the other hand, methylation of viral genomes might result in higher mutation rate at such positions, eventually reducing the amount of CpG dinucleotides [56,57]. Thus the different methylation patterns of novel host cells could also be responsible for the decrease in the overall G+C content of these viral genomes. The low G+C proportion in the sequence of the AmAdV-1 and LaAdV-1 genome fragments suggests that these viruses are likely to have switched hosts. The lack of disease or any clinical signs seemingly contradicts to this assumption, as generally an elevated pathogenicity is attributed to AdVs infecting a novel host. However, as the time of the eventual host switch events cannot be determined, and several individuals were found to carry the respective AdVs, we might assume a longer period, during which a transition from acute to persistent infection might have taken place. The position of AmAdV-1 on both phylogenetic trees, next to DAdV-1, might imply a host switch from a more remote, perhaps avian, host. This is also supported by the short length of the derived pX, in contrast with reptilian atadenoviruses, where it always exceeds 80 aa (84 aa for snake AdV-1 and 88 aa for lizard AdV-2) and more similar to that of non-reptilian atadenoviruses (67 aa in DAdV-1) [34,51,58]. It is also likely that LaAdV-1 originates from another, closely related lacertid species, hence infection of the Iberian green lizards happens sporadically, for example in the event of immunosuppressive effects. In case of Carpetane rock lizards, elevated testosterone levels during mating season were found to be in a positive correlation with apicomplexan parasite burden, suggesting testosterone to be a significant immunosuppressant [59]. Such immunosuppressive mechanisms, as both Iberian green lizard males displayed mating behavior, might have contributed to excessive shedding of AdVs via the gastrointestinal tract, hence facilitating the detection of viral DNA in the cloacal swabs screened in our work. LaAdV-2 appears more likely to have coevolved with the Carpetane rock lizard for a longer period of time. This scenario is supported, besides the balanced G+C content, by the existence of multiple genotypes as well.

## 5. Conclusions

Examinations aiming at the assessment of the prevalence and diversity of AdVs in free-living reptiles in natural reserves resulted in the discovery of three novel squamate AdVs in Spain. As the first AdVs ever detected in amphisbaenians and lacertids, their significance, regarding adenoviral diversity, is indisputable. Moreover, the presumed evolutionary background of AmAdV-1 and LaAdV-1 suggests that atadenoviral evolution might be more complex than originally thought. We revealed the possibility of multiple host switches between squamate reptiles and other vertebrates, as well as between squamates of different taxonomical status. Even though the detected AdVs did not appear to be responsible for any clinical signs or diseases in these endangered and/or highly protected reptile species, their prevalence in Iberian worm lizards and Carpetane rock lizards was found to be rather high. It is well known that subclinical persistent AdV infection might contribute to higher susceptibility for various infectious agents including parasites, bacteria or other viruses [36,60]. The uneven AdV prevalence among the animals belonging to different squamate species claims also attention, as interactions between these populations, due to habitat loss, as well as to the introduction of invasive species, could also lead to emerging diseases. Finally, the lack of positive cases among the reptiles, collected on the Chafarinas Islands, draws the attention to the importance of vigilance regarding the introduction of native or invasive reptiles. The spectrum of species sampled on the two study sites did not overlap at all, therefore it would be hard to decide if the AdV negative status of the Chafarinas Islands is real, and if so, whether it is due to the long-term isolation or some other conditions. Nevertheless, from a conservation perspective we definitely consider the first aspect to be plausible.
